# Supplementary material for: Are carbon emissions trading and green financial instruments synergistic? -Comprehensive quantitative research based on content analysis
Source: PLoS One. 2024 Mar 7;19(3):e0298601. doi: 10.1371/journal.pone.0298601 (PMC10919601; doi:10.1371/journal.pone.0298601)
Supplement: S1 Data — (ZIP) [file pone.0298601.s001.zip › Data/地级市Z_F工作报告.zip!/╝¬┴╓/╦─╞╜╩╨/└┤╫╘╣1⁄2╓┌║┼í╛┬φ┐╦╩2╛▌═°í┐_2015.docx]

各位代表：

　　现在，我代表市人民政府，向大会报告政府工作，请予审议，并请市政协各位委员提出意见。

　　一、 2014年工作回顾

　　刚刚过去的一年，在省委、省政府和市委的坚强领导下，在市人大、市政协监督与支持下，我们团结和依靠全市人民，深入实施“一核三带”富民优先战略，全面落实市委“五并重、一加强”、打造“五个强市”的部署要求，启动全面深化改革和法治政府建设，着力稳增长、调结构、促改革、惠民生、保稳定、强作风，较好完成了市七届人大二次会议确定的目标任务。

　　过去的一年，我们积极应对经济下行压力，主动作为，真抓实干，保持了经济健康平稳发展。预计全市地区生产总值实现 1310亿元，增长 8%；社会消费品零售总额实现 511亿元，增长 13.7%；全口径财政收入突破 100亿元，增长 5.2%；市本级全口径财政收入完成 56.2亿元，增长 7%。

　　过去的一年，我们协同推进新型城镇化和新农村建设，城乡统筹发展步伐不断加快。宜居宜业魅力新城的蓝图正加速变为现实，城市承载能力持续增强，“四梨同城化”取得实质进展。乡镇村屯面貌得到较大改观，形成农村人居环境建设“四平模式”。

　　过去的一年，我们注重把发展成果更多惠及百姓，年初承诺的 35件民生实事全面兑现。预计城镇居民人均可支配收入超过 2.6万元，农民人均纯收入有望突破 1万元。

　　过去的一年，我们重点抓了以下工作：

　　不断壮大经济规模。突出重大项目建设，培育新的经济增长点。预计完成固定资产投资 800亿元，增长 18%；新建续建亿元以上项目 281个、 10亿元以上项目 26个，“ 4· 18”集中开工的 47个亿元以上项目加速推进。烟厂年产 30万大箱卷烟易地改造、双辽迎新玻璃一期、梨树年产 6000吨木糖及 1000吨阿拉伯糖、红嘴开发区艾斯克禽类自动屠宰生产线等重点项目竣工投产，公主岭 300万辆汽车物流、梨树新希望集团年产 30万吨饲料、伊通吉林亿通商贸物流城、铁东万达城市综合体、铁西宝泰黄金珠宝城、辽河垦区百万台玉米播种器、红嘴开发区东风年产 3000台大型联合收割机等项目进展顺利。围绕国家振兴东北老工业基地政策谋划重大项目 137个。实施高层次、小分队、专业化招商，先后与 40余家战略投资者深入对接，引进 3000万元以上项目 688个，到位资金 668.8亿元。工业增加值实现 600亿元，增长 6.2%。与金融机构合作，成功发行二期 13亿元城投债，完成保障房一期 23亿元贷款，华利农业、建日生物、汶龙文化、韩州酒业等 7户企业在上海股权交易中心挂牌，年年文化、嘉德商场在吉林投交所挂牌。

　　持续推进产业升级。支持企业嫁接改造、科技创新，盘活存量资产，加快产业转型升级。百威英博全资收购金士百，铁东一汽四平专用车与中集东岳签订合作协议，梨树现代天丰与广药王老吉实现品牌合作，铁东中电建线路器材厂改造升级，红嘴开发区四平英力建机与柳工欧维姆合作打造中国北方预应力机具制造基地。省创新型科技企业达到 17户，省级技术中心 48家。实施技改项目 287项，完成投资 286.2亿元。工业首次投产新产品 141种，新产品产值率达 14.3%。巨元换热器、蓝宝石基板、军工刃具等产品行销国内市场，中国驰名商标达到 11件。四平市成为国家装备制造业新型工业化产业示范基地、国家第一批产业集群区域品牌建设试点城市、国家城区老工业区搬迁改造试点城市、“全国质量强市示范城市”创建市。省规划管理特色工业园区达到 11个，专用车、食品、换热器、机械装备、化工、 汽车零部件 6个产业列入省重点产业集群。大力发展现代服务业。完成商域港发展规划编制，电子商务产业园正式运营，与亿赞普公司签订战略合作和股权投资协议。铁西九洲第五郡特色商业街区投入运营，万嘉购物广场等服务业项目开工建设。突出发展民营经济，完善政策，设立基金，实施全民创业、招商引资、素质提升、集群发展和市场培育五大工程。预计全市民营经济主营业务收入实现 2220亿元，增长 9%；上缴税金 51亿元，增长 7.1%。

　　加快发展农村经济。围绕“三区一建”，全面落实惠农政策，着力提高农业综合生产能力。新建粮食高产示范区 100个，落实“高光效”新型栽培技术示范面积 96.7万亩，建立“农业专家会商机制”，启动“惠农通”农业信息平台。全市粮食产量达到 154.1亿斤，连续三年成为“全国粮食生产先进市”，被国家粮食协会命名为全国唯一的“中国优质玉米之都”。新建扩建棚室蔬菜园区 33个，新增生产面积 9080亩。新建扩建牧业小区 291个 ,带动全市畜牧业强劲发展。全市绿色食品企业发展到 16家，绿色产品 36个，“公主岭大米”获地理标志证明商标。大力发展多种形式适度规模经营，农民合作社发展到 9500个，家庭农场发展到 1218户，农村土地流转面积占承包地面积 25.7%。全力实施“农村人居环境建设行动计划”，建设“美丽乡村” 75个，打造 13条精品线路和 5个样板镇群。县域经济竞相发展。公主岭市积极推进扩权强县改革试点，县域发展水平居全省第二位，综合实力进入全国百强。梨树县 6户企业在上海股权交易中心挂牌，在开创资本市场上取得突破。双辽市依托产业基础和资源禀赋，谋划建成一批能够拉动县域经济快速发展的大项目。伊通县大力开发旅游资源，伊通镇被评为全国特色景观旅游名镇。辽河垦区强化商贸服务功能，孤家子镇被确定为省级商贸特色镇和扩权强镇试点镇。

　　统筹推进城乡建设。“四梨同城化”战略正式纳入省新型城镇化规划，户籍、教育、医疗等 8项便民利民实事得到落实。范家屯、郭家店、营城子、孤家子、 叶赫等 12个镇被列为省级重点 小城镇。东南生态新城紫昕广场全面竣工，法检两院正式入驻，城市规划馆主体封闭，占地 1000亩植物园开工建设。城区新建、翻建、维修道路 60条，新装、维修路灯 770组，改造升级广场 5座。高速公路、国省干线、农村公路建设同步推进，军民合用机场项目与沈空正式签订协议。“两河四岸”一期工程全面竣工，北河生态补水工程和北河公园改造已经完成，西湖湿地开工建设。市区和梨树县污水处理厂完成升级改造，市区淘汰 152台洗浴燃煤小锅炉，新建 107座水冲公厕。社区环境整治一期工程完工， 30个社区环境整体提升。大力推进“四绿工程”，全年造林 24万亩，绿树进城 43.8万株，市区绿化覆盖率达到 37.9%，全市森林覆盖率提高到 19.1%，四平市成为第一批国家生态文明先行示范区。

　　千方百计改善民生。新增财力重点投向民生领域，倾力办好民生实事。大力推进就业创业，全市城镇新增就业 4.24万人，城镇登记失业率控制在 4%以内，农村劳动力转移就业 42万人。进一步完善社会保障制度，城乡居民养老保险制度得到衔接，养老、医疗保险全覆盖，城乡低保和农村五保供养标准再次提高。改扩建养老院 20所，新建 10个社区日间照料中心。加大市区地下管网改造力度，新建改造供热管网 95公里，全面完成 15年以上老旧燃气管网改造任务。扎实推进保障性安居工程，市区新建廉租房 1.3万平方米，完成“暖房子” 225万平方米，改造棚户区 46万平方米，建设回迁安置房 56万平方米。完成城区二次供水改造一期工程，受益人口 17万人。建设农村饮水安全工程 39处，解决 3.7万农村居民、学校师生饮水不安全问题。实施“公交便民”工程，完善城区公交站点网络。采取集中执法、综合施策等措施，彻底解决 6000余辆三轮车非法上路营运问题，市区交通秩序明显改观。积极开展“市民文化节”、“农民文化活动月”活动，二龙湖、叶赫那拉城等 4个景区升级为国家 4A级景区。全民健身活动广泛开展，竞技体育在青奥会、亚运会、省运会取得优异成绩。加快教育事业发展，医护卫校、城建中专并入四平职大，辽北大学筹建工作进展顺利。建设标准化村卫生室 258个。完成省免费孕前优生健康检查项目任务。启动实施食品药品安全“三大放心工程”，查办违法案件 858件。不断完善社会治安防控体系，市区建成公安高清视频监控探头 839个，加强巡逻防控，社会治安明显好转。着力解决信访突出问题，呈现总量、来信、来访、非正常访下降和信访秩序好转态势。四平市被省政府确定为全省安全监管防控体系建设和国家安全生产综合改革试点城市，并召开了全省现场推进会。全年未发生较大以上安全生产事故，连续三年获得省政府目标责任制考核一等奖。

　　全面启动深化改革。按照市委统一部署，扎实推进重点领域和关键环节改革。市县政府机构改革全面完成，事业单位分类已经结束。市本级行政审批项目下降 31.1%。推进商事登记制度改革，实施“先照后证”等措施，新登记设立各类企业 3299户，增长 45.3%。农村土地确权登记试点进展顺利，公主岭市整体推进试点，其它县（市）区 11个试点村完成土地实测。土地收益保证贷款经验全国推广，被确定为全省林权、草权、水权收益保证贷款试点地区。村镇银行、农村资金互助社工作继续走在全省前列，两家“粮食银行”试点企业挂牌运营。全省唯一地级供销合作社综合改革试点顺利推进。县级医疗机构改革启动实施。

　　着力提升政府效能。按照中央、省委、市委统一部署，扎实开展党的群众路线教育实践活动，认真落实中央八项规定精神，严肃惩治腐败，“四风”问题得到有效遏制。落实 31项专项整治任务，全面清理超标超配公车和办公用房，“三公”经费大幅下降，作风建设得到加强。自觉接受人大、政协监督，办理人大代表建议意见 90件，办理政协委员提案 158件，办复率 100%。出台《四平市行政执法规定》，进一步规范行政执法程序和行为。成立政府法律顾问团和审计咨询顾问团，为政府决策和解决历史遗留问题提供支撑。扎实推进政务公开，市本级发布政府信息 8692条，市政务大厅审批事项即办件率达到 84%。坚持“问政于民、职责公开”，建立市、区、社区三级网络问政体系，解决了一批群众反映的困难和问题。国防动员和双拥共建深入开展。妇女儿童、民族宗教、广播电视、知识产权、外事侨务、人民防空、地震监测、通信、气象、档案、老龄、地方志、残疾人等事业取得新成绩。

　　各位代表！过去一年取得的成绩来之不易。这是市委科学决策、正确领导的结果，是市人大、市政协及社会各界有效监督、鼎力支持的结果，是全市上下团结拼搏、砥砺奋进的结果。在此，我代表市政府，向全市人民，向人大代表、政协委员，向各民主党派、工商联、无党派人士和人民团体，向驻平人民解放军、武警官兵和政法干警，向中省直驻平各单位和关心支持四平发展的国内外朋友，表示崇高的敬意和衷心的感谢！

在肯定成绩的同时，我们也必须清醒地看到存在的问题和差距。主要是：经济下行压力持续加大，一些骨干企业生产经营困难，部分重点项目推进缓慢，投资质量和效益还不高，财政收支形势异常严峻，经济增长的内生动力仍然不强；产业结构不尽合理，现代服务业发展滞后，创新能力不强，存量资产不活，稳增长、调结构、促升级的任务十分繁重；改革开放意识不够强，依法治市水平不够高，运用法治思维和法治方式处理问题还有差距；民生改善和保障还不够到位，公共服务质量还有待提升，一些群众利益诉求没有得到妥善解决；社会不安定因素依然存在，社会治理能力建设仍需强化， 维护社会和谐稳定的任务还比较艰巨；政府自身建设还不能完全适应新常态要求，部分干部作风漂浮，存在为官不为现象，“四风”问题还没有彻底根除，违法违纪案件仍时有发生。对这些问题和差距，我们必须高度重视，采取有效措施，切实加以解决。

二、 2015年工作安排

　　2015年是全面深化改革、建设法治政府的关键之年，也是“十二五”规划的收官之年。我们既面临着国家加快经济结构战略性调整的难得历史机遇，更有实施新一轮振兴东北老工业基地带来的重大政策利好。面对经济发展新常态，我们更要坚持把发展作为解决一切问题的关键，做到主动适应、尊重规律、创新驱动、注重效益，奋力开创四平发展振兴新局面。

　　2015年政府工作的总体要求是： 认真贯彻党的十八大和十八届三中、四中全会精神，全面落实省委十届四次全会和市委六届五次全会精神，按照发挥“五个优势”、推进“五项举措”、加快“五大发展”要求，围绕实施“一核三带”富民优先战略，推动“五个强市”建设，全面深化改革，建设法治政府，坚持稳中求进、好中求快、开拓创新、率先跨越，努力实现更高质量、更好效益、更美生态、更惠民生的发展，推进四平赶超隆起、全面振兴迈出坚实步伐。

　　经济社会发展的主要预期目标是：全市地区生产总值增长 7.5%，全口径财政收入增长 5%，固定资产投资增长 15%，工业增加值增长 8%，社会消费品零售总额增长 10%，城镇居民人均可支配收入和农民人均纯收入分别增长 7.5%。居民消费价格指数涨幅控制在 3.5%左右，城镇登记失业率控制在 4.5%以内。万元 GDP能耗下降 1.8%。

　　市委六届五次全会绘就了今后一个时期四平改革发展蓝图，这就是新一年政府施政的总纲领和总目标。我们必须进一步提高执行力，瞄准目标不动摇，心无旁骛抓落实，把已经确定的重大决策、重大项目、重大工程和重点工作紧紧抓在手上，积极有效作为，确保市委各项决策部署落地生根、结出硕果。重点抓好以下七个方面改革发展任务。

　　（一）突出投资拉动，保持经济持续稳定发展。调整优化投资结构，拉动经济快速增长，有效增添发展后劲。全年计划建设亿元以上项目 180个。全力攻坚重点项目。围绕“五个重大”，以扩大有效投资规模、增加财税贡献率、提高投入产出比为重点，完善重大项目考核办法，强化项目绩效评价，推动项目建设量质并举。抢抓国家新一轮支持东北振兴难得机遇，谋划推进 32个市级领导包保项目，加快史密斯专用车、君乐宝乳业奶制品加工、豪德商贸物流等一批重大项目的前期工作。积极推进军民合用机场、集双高速四平段、四平至通辽铁路电气化改造等项目。特别是围绕“ 4· 18”项目集中开工，抓好梨树绿色食品产业园、双辽银河真空玻璃及玻璃深加工、铁东文化广场综合改造、铁西君汇能源成品油仓储、红嘴帝玛生物年产 6000吨果蔬饮品等 30个以上亿元项目，抓紧完备规划、土地、环评等相关手续。推进工业存量提质增效。坚持工业立市不动摇，高度重视现有企业扩能改造，切实提高现有企业层级和竞争力。工业技改投资增长 8%。加快推进质量强市建设，实现质量升级，打造四平品牌。抓产需衔接服务，以东北亚博览会为展销平台，大力推销换热设备、专用汽车、鼓风机、收割机、家禽屠宰加工设备等一批中高端产品；抓银企对接服务，完善担保体系，做强助保金池，做好吉春制药、艾斯克、通成铝业等 7户企业在多层次资本市场上市工作，破解中小企业融资难题；抓运行协调服务，组织开展生产要素衔接，着力解决能源、运输等问题；抓包保服务，继续组织开展市县领导包保重点企业、万名干部服务万企等服务企业“大篷车”活动，引导企业嫁接改造，建设一批带动引领型项目；抓升级服务，支持中小企业建设新项目，创建创业孵化基地，实施《新增规模以上工业企业计划》，推动企业升级入规。突出发展民营经济。全面落实已出台的支持民营经济发展意见，制定民营企业进入特许经营领域具体办法，有效发挥专项资金引导作用，鼓励中小微企业向“专精特新”方向发展，扶持扩张一批旗舰型民营企业。全市民营经济主营业务收入计划实现 1749亿元，增长 10%；私营企业达到 1.1万户，增长 6%；个体工商业户达到 11万户，增长 7%。加强企业家队伍建设。大力营造尊重和关爱企业家的社会氛围，在政治上给地位、服务上给保障，实施“企业家素质提升”工程，开展法人治理结构升级试点，培育一支高素质的具有四平特色的企业家队伍。努力打造健康政商关系，在主动热情服务基础上，引导企业家诚实守信、依法经营，督促企业家照章纳税、履行义务，形成以效益分高下、以贡献论英雄的社会共识。

　　主动适应经济发展新常态，把握战略机遇，挖掘资源潜力，选准功能定位，启动“十三五”规划编制工作。着力谋划一批重大项目，为赶超隆起、发展振兴提供支撑。

　　（二）突出创新转型，加快产业结构优化升级。按照打造“五个强市”，构建“ 3+5”产业板块的总体布局，培育形成核心竞争优势。做大做强主导产业。推进四平市 8个国家级平台试点，引导企业靠大联强、嫁接改造、重组升级，抓好产业集群，建设好特色产业园区。专用车产业园，整合激活专用车资质资源，鼓励中航奋进建设房车、清淤车、清雪车等高端专用车，积极发展油田修井车，推进新能源汽车尽快落地，改造提升四平专用车产业基地。换热器产业园，完成国家换热器设备集群区域品牌试点示范市验收，打造公共平台完善、高端项目引领的中国换热器城。现代农机装备产业园，依托中科重机、康达农机和东风农机装备，大力发展谷王系列收割机、免耕播种机和大马力玉米收割机，打造农机研发产销基地。电力装备产业园，抓好线路器材厂易地提升改造，积极承接优质项目，力促电力装备产业园早日建成。通用航空产业园，积极推进通航产品制造基地和专属服务基地建设。玻璃建材产业园，加快双辽超白超薄屏显基板生产线等项目建设，形成枝繁叶茂的产业树。玉米深加工产业园，加快铁东天成 100万吨玉米加工项目建设，推动产业链条深度拓展。医药健康产业园，支持促进吉春大清鹿苑项目尽快落地，加快梅花鹿产业化步伐。生物化工产业园，加快选址规划审批进程，确保取得实质性进展。加快发展能源产业，重点推进 双辽庆达光伏生态产业园建设，加快伊通石油和梨树、双辽天然气资源的开发利用，构筑“四电一油气”产业格局。加快开发区和工业集中区基础设施建设，促进土地集约节约利用，促进开发区产业结构调整，大力发展新型工业和现代服务业，力争每个工业集中区建成一个省级县域产业转型升级示范区。切实把给予开发区的政策全部落实和衔接到位，真正使开发区成为领跑全市经济发展的“火车头”。突出科技创新驱动。加速推进四平市高新技术特色产业园区和吉林大学国家科技园四平园建设，力争新建院士工作站 1-2个、新建吉林省中试中心 1-2个。加大重点科技创新项目挖掘力度，力争 1-2个重点高新技术项目列入省“双十”工程。创新合作机制，开展好“院士专家四平行”活动，继续加强与吉林大学，吉林师范大学，中科院长春光机所、应化所、地理所等高校和科研机构合作，力争实现 3-5个产学研合作项目在四平落地。大力培育高新技术企业和省级科技企业，力争新增高新技术企业 1-2户、省级科技企业 10户。加快发展现代服务业。推动传统商贸提档升级，大力发展电子商务等新兴产业。全力推进万达城市综合体、宝泰黄金珠宝城、万嘉购物广场、总部展销城、东北区域总部服务会馆规划建设。继续完善电子商务产业园，推进亿赞普中国－东北亚跨境电子商务平台、国富商通大宗商品交易中心、阿里巴巴四平农业产业带、传化智能公路港、四平吉高物流甩挂运输物流园等项目建设，创建国家级电子商务示范基地。丰富和完善四平战役纪念馆陈列内容，加大历史文化街区以及叶赫、山门等重点旅游景区建设力度，加快推进今日集团百花欢乐大世界建设，带动经济转型升级。

　　（三）突出“三区一建”，加快发展农业农村经济。坚持用改革创新统领“三农”工作，努力实现粮食增产、农业增效、农民增收和民生改善的目标。全面提升农业综合生产能力。稳定粮食作物面积，建设粮食高产示范区 75个。加快新技术、新品种集成推广应用，实现测土配方施肥、玉米螟常发区生物防治技术全覆盖。抓好国家新增千亿斤粮食工程，完善地方粮食收储保障体系，稳步促进粮食丰产增收。加强农业基础设施建设，增加和改善节水灌溉规模，提高农业机械化水平，农作物耕种收割综合机械化水平达到 77%。加强标准化牧业小区建设，不断壮大畜禽饲养规模，提高疫病防控能力。推进规模化棚室园区建设，新建扩建棚室蔬菜园区 10个，新增棚室面积 4000亩。进一步提升农业产业化发展水平，夯实现代农业发展产业基础。加快发展绿色食品产业。打造国家食源性绿色安全农产品基地，抓好特色现代农业试验区和示范区建设，发挥“中国优质玉米之都”优势，大力发展品牌农业、精品农业、生态休闲观光农业，适时举办梨树绿色食品节，主推辽河大米和梅花鹿制品，新认证的 “三品一标 ”产品和绿色食品产值均比上年增长 10%以上。深化农村经营机制创新。在防止农村土地非农化基础上，大力推进农村土地经营权规范流转，积极培育家庭农场等新型农业经营主体和多元农业社会化服务主体。利用省级扶持资金，抓好 95个村集体经济经营方式创新。推进财政支持农民合作社创新试点 ,引导发展农民专业合作社联合社。依托农村“三资”代理服务中心，完善和完成县、乡两级农村土地流转服务中心和农村产权交易中心建设试点任务。强力推进美丽乡村建设。启动农村新型社区和美丽乡村建设模式试点，建立健全农村环境建设投入机制、共建机制、长效管护机制，全面实施农村环境清洁、基础设施建设、人畜分离、垃圾污水处理、公共服务配套和生态环境改善“六大工程”，重点完成 30项整治建设任务，以节点镇为依托，力争全市再有 40个村达到“美丽乡村”标准，每个县（市）区打造 2－ 3个集中连线的新农村精品线路。

　　（四）突出扩容提质，推动特色新型城镇化建设。积极创新服务管理方式，加大投入解决突出问题，让城乡更加宜居、社会更加有序、生活更加便利。加速建设新城。积极完成东南生态新城道路、桥涵等基础设施新建续建工程，加快城市规划馆布展工作，开工建设体育馆、文化艺术中心等重点工程，加快建设 1000亩植物园。改造提升主城。加快城区路网建设，完成紫气大路西段等重点道路工程，建好棚改地块周边 15条道路以及师院北街等 22条断头路和巷路，逐步形成十三横十二纵主次路网格局。研究推进市区 14个城中村改造，完成剩余 46个社区环境优化整治，实施弃管小区物业管理新模式。狠抓巷路、步行街、排水管网、垃圾中转站等基础设施建设与管护，加大道路清扫机械设备投入，道路机扫率达到 85%以上。优化生态环境。开工建设南、北河污水处理厂和郭家店污水处理厂，进一步淘汰 10蒸吨及以下燃煤小锅炉。推进双辽国家级白鹤自然保护区申报进程。加快西湖湿地建设，完成南北河再生水利用工程。做好转山湖水库应急引水、灌区节水改造、小型农村水利等重点工程前期工作。继续实施造林绿化，完成造林 15万亩，提升城市绿化美化标准。打造“智慧四平”。突出政府引导、市场主导作用，推进信息惠民试点工作，完善 NFC手机市民卡、公共民生等智慧城市硬件平台建设。推进县镇建设。抓紧编制“四梨同城化”详细规划，在重大基础设施和便民利民实事上取得突破性进展。加快重点城镇规划编制，扎实推进梨树、伊通、双辽新城建设。加快推动辽河垦区行政区划调整，解决制约发展问题。抓好公路建设。抓紧舍开路项目筹资，加快工程建设步伐。完成四平出口互通立交桥建设，确保长平、长双高速竣工通车。新（改）建农村公路 200公里。

　　（五）突出百姓福祉，全力办好各项民生实事。坚持发展成果惠及百姓，努力提高全市人民的幸福感和满意度。积极促进就业增收。推进城镇稳定就业人员和劳务输出人员实名制，以市区街道、社区和村屯为重点，构建零距离就业服务圈，实现就业服务信息全覆盖。完善工资集体协商制度，健全企业职工工资正常增长和支付保障机制，适时调整最低工资标准。按政策标准提高机关事业单位人员补贴。落实基础保障工程。市区棚户区改造建设回迁安置用房 80万平方米，建成廉租住房 4万平方米，完成“暖房子”改造 160万平方米，新建改造供热管网 29.5公里，新建换热站 15座，改造老旧燃气管网 35公里，铺设燃气管线 30公里，完成市区二次供水改造和农村饮水安全建设项目 。建设南环 2万平方米城市公交综合保障中心，继续新开公交线路、新增公交车辆。提升公共服务质量。切实做好社会保障工作，全面排查未参保企业和已参保企业的未参保职工，尽最大努力应保尽保。设立 统一受理社会救助服务窗口，落实城乡低保自然增长机制。加强标准化幼儿园建设， 大力推进义务教育均衡发展，深入实施四平盲童学校省市共建项目，进一步整合职业教育资源，推进集团化办学，继续做好组建辽北大学工作。扎实推进基本公共卫生服务，完善省内异地就医即时结算平台，全面完成村卫生室建设任务。坚持计划生育基本国策，落实单独两孩生育政策。坚持文化“七进”、“三下乡”，继续推进全民阅读活动，探索提供公共文化服务新模式。广泛开展全民健身活动，力争城市社区和两区乡镇、行政村体育健身器材全覆盖。积极承办全国体育赛事，打造四平体育赛事品牌。支持社会力量兴办养老服务机构，市社会福利院改造工程主体封闭。提升食品药品安全检验检测能力，深化食品药品安全专项整治，确保公众饮食用药安全。

　　（六）突出改革开放，真正激活经济发展动力。按照中央和省委、市委部署，落实全面深化改革具体任务，构建多层次开放格局，激活土地资源、创业主体和资本市场，尽快适应经济发展新常态。进一步简政放权。继续推进行政审批制度改革，逐步取消非行政许可审批项目。深化政府机构改革，加快事业单位分类改革，推进行业协会、商会与行政机关脱钩。深化农村土地制度改革。突出抓好 21个乡镇农村土地承包经营权登记确权颁证工作。稳妥推进市场化改革。实行市场主体经营异常名录制度，公开许可登记管理信息，抓好土地、林草、水面等领域物权融资改革试点，推进供销系统综合改革试点工作。推动社会治理体制改革。落实招生考试制度改革，做好户籍制度改革相关配套工作，开展县级公立医院综合改革，适时推进机关事业单位养老保险制度改革，实行城乡统一、可转移接续的社会保险制度。积极支持公主岭市扩权强县试点。整体推进管理体制改革，促进县域经济快速发展。全方位扩大对外开放。 继续 坚持高层次、小分队、专业化、产业链招商，瞄准装备制造、农产品加工及高端服务业等领域， 择商选资、招大引强。利用东博会、全国工商联“一会一活动”、汉诺威工业博览会等平台推介项目。推进与美国杜邦、德国史密斯和克拉斯、荷兰帝斯曼等企业深度洽谈。促成梨树台湾食品园、铁东金隅建材产业园、中电建高分子管道等项目签约。推动双辽博来德内陆港与大连、丹东、营口等港口合作。

　　（七）突出安定和谐，切实提升社会治理能力。发挥政府主导作用，不断创新社会治理方式，努力构建安居乐业美好家园。加强基层组织管理。坚持社会管理重心下移，推进落实“社区行动计划”，实行社区公共服务事项准入制度，建立和完善村民民主听证、咨询等制度，推进基层民主协商工作。深化平安四平建设。加强社会治安综合治理防控体系建设，全面开展平安创建活动，建立实有人口服务管理机制，加大交通、消防和危险物品安全隐患的整治力度，依法打击各类违法犯罪活动，加大巡逻防控，净化治安环境，市区、县域重点部位视频防控覆盖率达到 100%。做好信访维稳工作。强化领导责任落实，健全重大决策社会稳定风险评估机制，从源头上预防和减少信访问题发生。做好接访下访工作，启动联合接访大厅建设，健全解决信访突出问题工作机制。加大矛盾纠纷调处力度，加强法律服务中心、法律咨询服务站、百姓说事点建设，不断扩大人民调解覆盖面。完善涉法涉诉信访依法终结制度，严格依法处置违法上访行为。落实安全生产责任。深入开展安全生产隐患排查治理，推进“四化融合”、“三位一体”安全监管防控体系改革试点工作，严格重点行业领域安全专项整治，建立健全部门联动机制，加强应急管理和值班值守，不断提高安全监管防控体系建设水平。深入开展双拥工作，努力争创全国双拥模范城“七连冠”。

三、加快法治政府、廉洁政府建设

　　认真落实市委关于全面推进依法治市和反腐倡廉的部署要求，坚持在法治轨道上开展工作，推进依法行政，打造法治政府、廉洁政府，全面提升政府工作整体水平。

　　依法全面履行职能。完善行政组织和行政程序法律制度，推进机构、职能、权限、程序、责任法定化。坚持法定职责必须为、法无授权不可为。加大政府信息公开力度，认真贯彻新《预算法》，推进财政预决算公开。加强政务大厅等平台建设，研究实施网上审批，让行政权力在阳光下运行。坚决纠正不作为、整治乱作为、问责无效作为，坚决克服懒政、怠政，坚决惩处失职、渎职。推进权力清单建设，坚决消除权力设租寻租空间。加快建设职能科学、权责法定、执法严明、公开公正、廉洁高效、守法诚信的法治政府。

　　健全依法决策机制。自觉接受党内监督、人大监督、民主监督、司法监督、审计监督、社会监督、舆论监督，形成科学有效的权力运行制约和监督体系。进一步完善政府法律顾问团制度，尽快建立专家咨询团，对重大决策事前合法审查、组织专家咨询。把公众参与、专家论证、风险评估、合法性审查、集体讨论决定作为重大行政决策法定程序，确保决策过程置于法律框架内运行。继续坚持政府重大决策听取咨政意见和听证制度，健全完善行政决策规则和程序，建立重大决策终身责任追究制度及责任倒查机制，避免和减少决策失误，提高决策质量。

　　严格规范行政执法。实施《四平市行政执法规定》，建立执法全过程记录制度，健全行政裁量权基准制度、行政执法和刑事司法相衔接制度，落实重大执法决定法制审核制和行政执法责任制，最大限度压缩自由裁量空间，强化行政执法监督，重点解决执法不规范、不文明等突出问题。全面开展普法教育，推动全社会树立法治意识。深化行政执法体制改革，健全完善市县两级执法体制，积极推进综合执法、跨部门执法，解决食品药品、安全生产、环境保护、城乡管理等领域突出问题，切实维护群众利益和经济社会秩序。

　　切实做到从严治政。全面落实 “一岗双责 ”，健全完善惩治和预防腐败体系，深化“五权”工作，坚持无禁区、全覆盖、零容忍，严厉查处腐败问题。全体政府公职人员都要严守党的政治纪律和政治规矩，做到“心中有党、心中有民、心中有责、心中有戒”，做党和人民的好干部、好公仆；都要心怀畏惧，抵制诱惑，严格执行中央八项规定和省委、市委具体规定，坚决防止“四风”问题反弹；都要敢于担当，勇于作为，依据法治谋赶超，尊重规律图振兴，努力干出经得起实践和时间检验的业绩；都要从全市根本利益出发，千方百计为企业和项目服务，刹歪风、树正气，创造良好的投资发展环境；都要注重调查研究，直面矛盾问题，强化督查问责，狠抓工作落实，务求取得实打实的工作成果。

　　各位代表！

　　新的一年，政府工作任务目标已经明确，唯有付出更大努力，才能担当起发展重任；唯有倾注全部心血，才能不负人民重托。让我们在市委坚强领导下，凝心聚力，真抓实干，开拓创新，为实现四平赶超隆起、全面振兴而努力奋斗！
